# Exploring the anticancer mechanism of cardiac glycosides using proteome integral solubility alteration approach

**DOI:** 10.1002/cam4.70252

**Published:** 2024-09-30

**Authors:** Wenjie Qin, Yinhua Deng, Huan Ren, Yanling Liu, Ling Liu, Wenhui Liu, Yuxi Zhao, Chen Li, Zhiling Yang

**Affiliations:** ^1^ Department of Pharmacy The First Affiliated Hospital of Hunan Normal University (Hunan Provincial People's Hospital) Changsha China; ^2^ Department of Pharmacy The Second Xiangya Hospital, Central South University Changsha China; ^3^ Institute of Clinical Pharmacy, Central South University Changsha China; ^4^ Shenzhen Wininnovate Bio‐Tech Co., Ltd Shenzhen China

**Keywords:** cardiac glycoside, digitoxin, digoxin, drug repositioning, drug repurposing, off‐target, ouabain, PISA

## Abstract

**Background and Aims:**

Cardiac glycosides (CGs), traditionally used for heart failure, have shown potential as anti‐cancer agents. This study aims to explore their multifaceted mechanisms in cancer cell biology using proteome integral solubility alteration (PISA), focusing on the interaction with key proteins implicated in cellular metabolism and mitochondrial function.

**Methods:**

We conducted lysate‐based and intact‐cell PISA assays on cancer cells treated with CGs (Digoxin, Digitoxin, Ouabain) to analyze protein solubility changes. This was followed by mass spectrometric analysis and bioinformatics to identify differentially soluble proteins (DSPs). Molecular docking simulations were performed to predict protein‐CG interactions. Public data including gene expression changes upon CG treatment were re‐analyzed for validation.

**Results:**

The PISA assays revealed CGs’ broad‐spectrum interactions, particularly affecting proteins like PKM2, ANXA2, SLC16A1, GOT2 and GLUD1. Molecular docking confirmed stable interactions between CGs and these DSPs. Re‐analysis of public data supported the impact of CGs on cancer metabolism and cell signaling pathways.

**Conclusion:**

Our findings suggest that CGs could be repurposed for cancer therapy by modulating cellular processes. The PISA data provide insights into the polypharmacological effects of CGs, warranting further exploration of their mechanisms and clinical potential.

## Graphical abstract

Cardiac glycosides (CGs) have garnered attention for their potential as novel antitumor drugs. In this study, we employed proteome integral solubility alteration (PISA) to explore the multifaceted mechanisms of action of CGs, including digoxin, digitoxin, and ouabain, within the context of cancer cell biology. Through both lysate‐based and intact‐cell PISA, we conducted a comprehensive analysis of protein interactions and solubility changes following CGs treatment, suggesting that CGs could be repurposed to target cancer through a multifaceted approach that modulates cellular metabolism, ribosomal function, and mitochondrial activity.

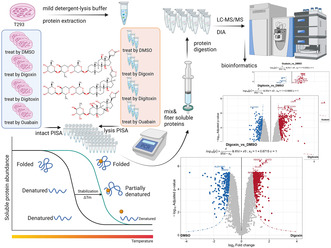

## 1 INTRODUCTION

Cancer is a critical global health issue with rising incidence and mortality rates.^1^, ^2^ The burden of this disease results in substantial economic costs and a vital need for innovative and affordable drugs.^3^ Traditional antitumor drug discovery strategies involves extensive preclinical and clinical studies, leading to high development costs and prolonged timelines. Despite substantial investments, the success rate for compounds entering clinical trials and reaching the market remains low, highlighting the necessity of simplifying the procedure for developing cancer treatments and addressing the astronomical costs that make them unaffordable, especially in underdeveloped countries.^4^

Drug repurposing, also known as drug repositioning, has emerged as a promising strategy for developing antitumor drugs.^5^, ^6^ Repositioned drugs, which include various chemotherapeutic agents, have demonstrated potential effects such as immunomodulation, antiproliferation, pro‐apoptosis, and metastasis inhibition. By applying old or structurally modified drugs to new indications, researchers can leverage existing data on the safety, toxicity, and pharmacological properties of these drugs. This approach facilitates a faster progression through preclinical and clinical trials, potentially leading to cost savings and shorter R&D cycles.^7^, ^8^

Since 1785, cardiac glycosides (CGs) extracted from the *Digitalis* plant have been used to treat congestive heart failure. Digoxin, a widely prescribed CG, is listed as an essential medicine by the World Health Organization and is one of the most prescribed naturally derived pharmaceutical products. CGs were first described as specific inhibitors of the Na+/K + ‐ATPase^9^ and are clinically used for heart diseases, including atrial fibrillation and heart failure. Recent studies have explored the potential of CGs in the treatment of other diseases, especially cancer, and its antitumor mechanisms include activation of Na+/K + ‐ ATPase, inhibition of DNA topoisomerase II, promotion of reactive oxygen species (ROS) production, disruption of glycolysis in tumor cells, etc.^10^ Studies have shown that different CGs have different effects on different types of tumors, and their targets have been newly reported one after another.^11^, ^12^ However, so far, the action of CGs on tumors as target sites and their mechanism of action still need to be investigated.

Chemical proteomics is a key tool in drug development, focusing on the study of drug target phenotype relationships.^13^, ^14^ Traditional chemical proteomics methods are limited by the use of chemically modified ligands, while newly developed and evaluated methods that do not require chemically modified ligands bring new possibilities to this field.^15^, ^16^, ^17^, ^18^, ^19^, ^20^, ^21^ Mass spectrometry (MS) coupled to the cellular thermal assay (CETSA), which is extended into thermal proteome profile (TPP), has become a popular technique for identifying drug targets and off‐targets by using ligand‐induced changes in the thermal stability of proteins.^16^, ^17^, ^22^, ^23^, ^24^, ^25^

To enhance analysis throughput and reduce the number of samples required, new formats of thermal assays such as proteome integral solubility alteration (PISA),^26^ isothermal shift assay (iTSA),^27^ and matrix thermal shift assay (mTSA)^28^ have been developed. Furthermore, data‐independent acquisition (DIA)‐based label‐free quantification approaches has compared to tandem mass tag (TMT) data‐dependent acquisition (DDA) for thermal shift or soluble difference quantitations.^18^, ^19^, ^21^ Utilizing label‐free methods to further enhance throughput and reduce costs, the advancements in next‐generation MS and DIA allow for surpassing the quantitative depth of traditional TMT DDA, while also ensuring statistical robustness.

Given that the exact target of CGs, such as Na+/K + ‐ATPase, is known and causes intracellular calcium ion influx as a possible source of disturbance, PISA with live cells and protein extracts can better determine the direct effects of other pathways. In this study, we employed the DIA‐based PISA approach to explore digoxin, digitoxin, and ouabain, aiming to comprehensively investigate their potential interaction targets and anticancer mechanisms.

## 2 MATERIALS AND METHODS

### 2.1 Chemical reagents

Digoxin (HY‐B1049), Digitoxin (HY‐B1357), and Ouabain (HY‐B0542) were all obtained from MedChemExpress (MCE, USA). All other commercially available chemicals, unless otherwise specified, were purchased as either pure synthesis or analytical grade reagents from Sigma‐Aldrich (St. Louis, MO, USA).

### 2.2 Cell viability assay

The human embryonic kidney cell line HEK293T (293T), the human breast cancer cell line MCF7, and the human lung adenocarcinoma cell line A549 were obtained from ATCC. All cells were cultured in DMEM (Gibco) supplemented with 10% FBS (fetal bovine serum), 100 μg/mL streptomycin, and 100 U/mL penicillin (Gibco) at 37°C with 5% CO_2_.

To evaluate the effect of digoxin, digoxin and ouabain on cell proliferation or cytotoxicity, referring to the literature,^29^, ^30^, ^31^, ^32^ 293T, A549, and MCF7 cells were treated with 40 nM digoxin, digoxin and ouabain for 0, 12, 24, and 36 h, respectively. The cell viability was evaluated using the Cell Counting Kit‐8 (CCK‐8) (TargetMol, USA) method according to the manufacturer's instruction.

### 2.3 Sample preparation for PISA

Samples for the PISA assay were prepared by referring to the previous study with minor modifications.^26^, ^33^ For lysate‐based PISA analysis, cells were lysed using a final concentration of 0.4% NP‐40, optimized for membrane protein solubilization,^24^ and incubated for 30 min at 4°C to ensure efficient extraction. Subsequently, they were incubated at 37°C with DMSO and 40 nM CGs (digoxin, digoxin, and ouabain) for 30 min, respectively, centrifuged and then transferred the supernatant into 0.2 mL PCR (polymerase chain reaction) tubes. Concurrently, two distinct PCR programs were initiated, each running for 3 min: (a) a temperature gradient program with temperatures ranging from 44°C to 52°C at the tenth step and (b) a different gradient ranging from 53°C to 60°C at the tenth step. It was then incubated at room temperature for 3 min and filtered with a 0.22‐μM filter to extract the soluble protein. For intact‐cell PISA, 293T cells were cultured in six‐well cell culture plates to 70% confluency, treated with medium containing DMSO and 40 nM CGs (digoxin, digoxin, and ouabain), respectively, for 30 min, harvested, and then washed three times with PBS (phosphate‐buffered solution) buffer. The resulting cell suspensions were carefully transferred into 0.2 mL PCR tubes. The above two PCR procedures were performed in parallel for 3 min. Following this, the cells underwent a brief incubation period of 3 min at room temperature. After incubation, the cells were carefully transferred into 1.5 mL EP tubes and lysed in a final concentration of 0.4% NP‐40 for 30 min at 4°C. Precipitated proteins were separated from the soluble fraction by 0.22 μM filter.

### 2.4 Protein digestion

Filter‐aided sample preparation (FASP) method was used for in‐solution digestion.^34^ Briefly, aliquots of proteins were mixed with 200 μL of 8 M urea in Nanosep centrifugal devices with Omega membrane 10 kDa (PALL, USA). The device was centrifuged at 12,000 × *g* at 20°C for 20 min. All the centrifugation steps were performed applying the same conditions allowing maximal concentration. Then, 200 μL of 8 M urea solution with 10 mM dithiothreitol were added, and the reduction reaction was allowed to proceed for 2 h at 37°C. The solution was removed by centrifugation, and 200 μL 8 M urea solution with 50 mM iodoacetamide was added. The sample was incubated in the dark for 15 min at room temperature. The ultra‐fraction tube was washed three times with 200 μL of 8 M urea and 200 μL of 25 mM ammonium bicarbonate by centrifugation at 12,000 × *g* for 20 min at room temperature. Then, 100 μL of 25 mM ammonium bicarbonate containing 0.01 μg/μL trypsin was added to each filter tube. The tubes were incubated at 37°C for 12 h. The filter tubes were washed twice with 100 μL of 25 mM ammonium bicarbonate by centrifugation at 12,000 × *g* for 10 min. The flow‐through fractions were collected and lyophilized.

### 2.5 Mass spectrometric analysis

The lyophilized peptide fractions were re‐suspended in ddH_2_O containing 0.1% formic acid, and 2 μL aliquots of which were loaded into a nanoViper C18 (Acclaim PepMap 100, 75 μm × 2 cm) trap column. The online chromatography separation was performed on the Easy nLC 1200 system (ThermoFisher, USA). The trapping, desalting procedures were carried out with a volume of 20 μL 100% solvent A (0.1% formic acid). Then, an elution gradient of 5%–38% solvent B (80% acetonitrile, 0.1% formic acid) in 120 min was used in an analytical column (Acclaim PepMap RSLC, 75 μm × 25 cm C18‐2 μm 100 Å). DIA mass spectrum techniques were used to acquire tandem MS data on a Q Exactive mass spectrometer (ThermoFisher, USA), equipped with a high‐sensitivity Nano Flex ion source. The primary MS scan has a resolution of 70,000, a scanning range of 350–1200 *m*/*z*, and a maximum injection time of 50 ms. Based on the density of mass peaks in the pre‐mixed sample, the data collection is set to a variable window, with 34 separated windows in each cycle, and an overlap of 1 Da between windows. The maximum injection time for the secondary MS ions is also 50 ms. The collision cell energy (high‐energy collision‐induced dissociation, HCD) is set to 28 eV.

### 2.6 Data processing and bioinformatics analysis

The MS/MS data were analyzed for protein identification and quantification using DIA‐NN 1.8.1^35^ against the Swiss‐Prot reference database of Homo sapiens containing nonredundant 20,396 proteins of 20,195 genes. In the DIA‐NN settings, default configurations with a slight modification of maximum number of variable modifications set to 2 with oxidation (M) and protein N‐term acetylation. Match between runs were set, which is beneficial for most quantitative experiments.

We employed R language (v4.3.2) and R package DEP2 (v0.5.4.02)^36^ for proteomics data analysis using protein quantities reported by DIA‐NN. Briefly, protein groups filtered base on missing number is < = 1 in at least one condition, log2‐transformed and further normalized using variance stabilizing transformation (VSN). MissForest missing value imputation used for better estimation on missing values.^37^ Hypothesis testing performs a moderated *t*‐test using limma with Benjamini–Hochberg (BH) FDR control method. We utilized a curve cut off approach as described by Eva C. Keilhauer^38^ to identify significant differentially soluble proteins (DSPs). This approach uses curve lines with *y* > *c*/(*x* − *x*_0_), where x is the log2 fold changes (L2FCs), y is the adjusted *p* values (pAdj) of DSPs. The parameters *c* and *x*_0_ represent the curvature and minimum L2FC, which set to 1 respectively. The value of *x*_0_ is determined using the standard deviation *σ* by *x*_0_ = *x*_0_.fold*σ. *σ* is the standard deviation of the Gaussian curve distribution of L2FCs in each contrast. The thermal stability score calculated with‐log10(pAdj) for DSPs in corresponding CGs versus DMSO.

Pathway and process enrichment and protein–protein interaction (PPI) analysis for DSPs was carried out using Metascape.^39^ For PPI analysis, only the physical interactions in STRING (physical score >0.132) and BioGridwereutilized as the default parameter of Metascape. Heatmaps were generated with the R package ComplexHeatmap (v2.18.0)^40^ or Morpheus (https://software.broadinstitute.org/morpheus).

### 2.7 Molecular docking simulations

The DSPs labeled in Figure 2 and in lysate‐based ouabain group were output for molecular docking simulations. CB‐Dock2^41^ were utilized for molecular docking and visualization. Protein structures using AlphaFold v4 prediction model^42^ and mature forms of GOT2 and GLUD1.^43^ The chemical structures of the three CGs were obtained from PubChem. The results of binding affinity were expressed as the Vina score, with lower scores representing higher binding affinities.

### 2.8 Reanalysis of DEGs in C4‐2B cells treated with digoxin

We used GEO2R (https://www.ncbi.nlm.nih.gov/geo/geo2r/) to reanalyze differentially expressed genes (DEGs) in prostate cancer cell lines treated with digoxin.^44^ C4‐2B cells treated with 100 nM digoxin for both 24 and 48 h, along with their corresponding vehicle treatments, were used for this analysis. A *p*‐value threshold of less than 0.1 was set to identify DEGs, resulting in the selection of 3580 genes for trend analysis. Data and GEO2R analysis are available in GSE230039 series of GEO database (http://www.ncbi.nlm.nih.gov/geo). We used the R package ClusterGVis (v0.1.1)^45^ to perform clustering and visualization of DEGs based on their transcripts per million (TPM) values across time‐series samples, The results from the Gene Ontology biological process (GOBP)^44^, ^46^ and Kyoto Encyclopedia of Genes and Genomes (KEGG)^47^ enrichment analyses, conducted using the clusterProfiler package (v4.10.0),^48^ were incorporated into ClusterGVis for further investigation.

## 3 RESULTS

### 3.1 Cell viability assays determined the effects of CGs on cells

**FIGURE 1:**
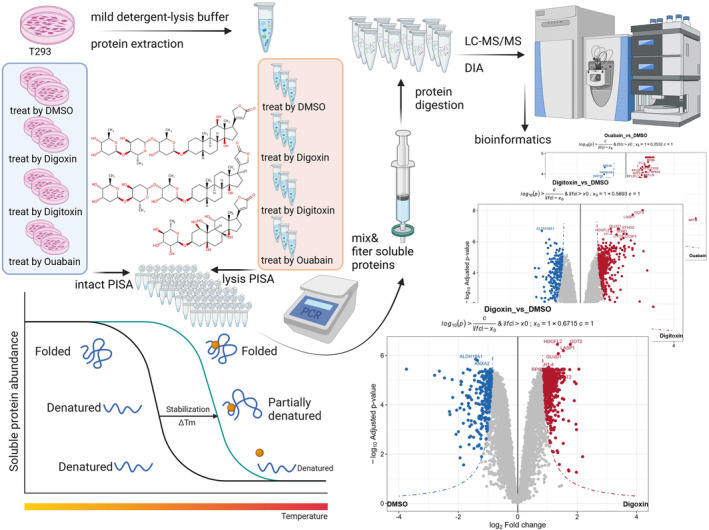
The scheme of PISA strategy.

To examine and determine the cytotoxicity and effects of CGs on cell proliferation, 293T cells (human embryonic kidney cell line HEK293T), MCF7 cells (the Human breast cancer cell line MCF7), and the A549 cells (human lung adenocarcinoma cell line A549) were selected for the study. Referring to the previous studies,^29^, ^30^, ^31^, ^32^ the action concentration of CGs (digoxin, digitoxin, and ouabain) was 40 nM. The results showed that these three types of CGs had inhibitory effects on all three types of cells, especially on 293T cells (Figure S1). Thus, we selected 40 nM as the action concentration and 293T cells as the study object, through the combined application of PISA and DIA MS, the global interaction of the three types of CG was evaluated based on the detection of intact cells and lysates. The analysis strategy and scheme are shown in Figure 1.

### 3.2 Lysate‐based PISA to identify CGs targets

**FIGURE 2:**
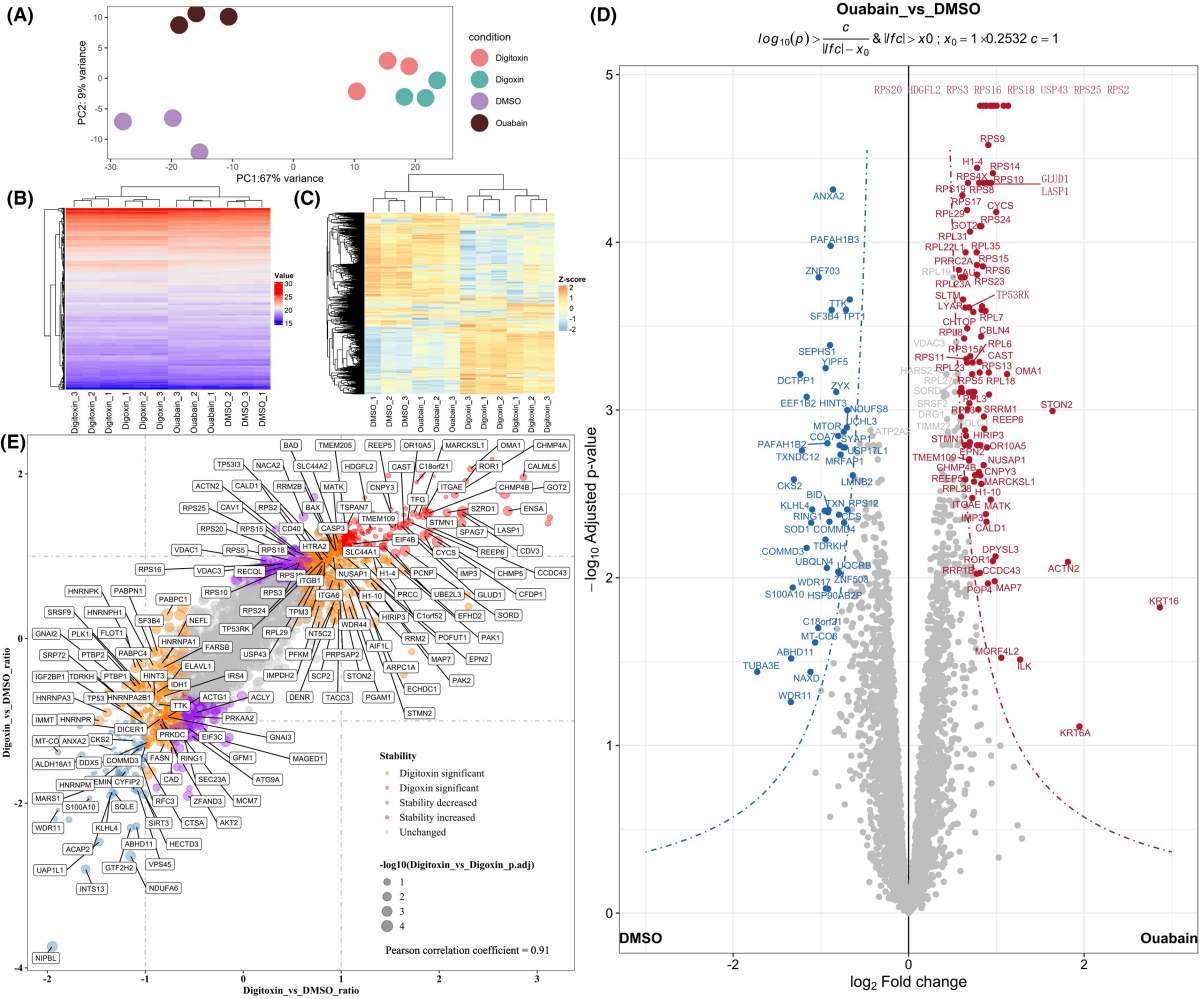
Lysate‐based PISA to identify CGs targets. (A) PCA analysis of lysate‐based PISA groups. (B) Heatmap combined with dendrogram to show clustering of samples by processed quantitative proteomics intensity values. (C) The heatmap shows the *z*‐scores of the processed quantitative proteomics data by row. (D) Volcano plots of ouabain versus DMSO, DSPs were highlighted and red for significantly increase in solubility change and blue for decrease. (E) Correlation analysis between digoxin and digitoxin, both increase in solubility change were colored by red, and both decrease in solubility change were colored by blue, and threshold of twofold change were set, then the DSPs in digitoxin and digoxin were sequentially colored by orange and purple. The bubble size corresponds to‐log10 of the adjusted *p*‐value from the comparison between digitoxin and digoxin.

Lysate‐based PISA assays were conducted to classify the primary direct ligand binding. The lysates of 293T cells were exposed to CGs (digoxin, digitoxin, and ouabain) and measured soluble amounts of proteins in the CG‐treated and DMSO‐treated samples (Figure 1). A total of 4088 proteins were identified, in which 4017 were unique proteins (Figure S2 and Table S1). Protein groups matrix only keep the first inferred protein for further analysis, 4070 filtered proteins were then normalized by variance stabilizing transformation (VSN) and missing values were imputed by random forest method (Figure S2 and Table S1). PCA (principal component analysis) was applied to evaluate the data quality by ensuring the consistency within the groups and separation between the groups. As shown in Figure 2A, the digoxin‐ and digitoxin‐treated groups are very close to each other, because they are very similar in structure. The DMSO‐ and ouabain‐treated groups were separated from each other, and they were also separated from the other two groups. Analogous global information was intuitively displayed by heatmaps (Figure 2B,C).

Changes in soluble protein levels and confidence scores were integrated using a cutoff curve method for comprehensive analysis and screening. We specifically accentuated the details of the ouabain group (ouabain/DMSO group) by volcano plot, revealing a substantial increase in ribosomal proteins, particularly those of the small subunit, which exhibited markedly lower adjusted *p*‐values (Figure 2D). The digoxin (digoxin/DMSO group) and digitoxin group (digitoxin/DMSO group) had more DSPs, which was consistent with the PCA and heatmap results (Figure 2A–C). This might be due to their more complex structures which are more likely to generate more interactions. A total of 594 DSPs were identified in the digoxin group, 690 DSPs in the digoxin group, and 143 DSPs in the ouabain group (Table S2). The proteins most affected by all three CGs were all significantly upregulated and included glycolytic enzyme GOT2, glutamate dehydrogenase GLUD1, hepatoma‐derived growth factor‐related protein HDGFL2, LIM and SH3 domain protein LASP1, cytochrome c CYCS, and de‐ubiquitinating enzyme USP43.

A strong correlation was observed between the digoxin and digitoxin groups (Figure 2E), which may be attributed to their highly similar structures. However, there were also significant differences between them, which may account for their differential actions. Interestingly, the kinases such as PAK1 and PAK2 displayed a unique increase in solubility in response to digitoxin treatment. Additionally, in the presence of digoxin and digitoxin, there was a sustained decrease in the solubility of TP53, along with a decrease in the response of MTOR to ouabain. The biophysical principle of ligand‐induced thermal stabilization of target proteins suggests that the binding of small molecules to proteins leads to an increase in stability. The correlation plot for digoxin and digitoxin also shows a clear trend, with a greater number of instances exhibiting increased stability rather than a reduction (Figure 2E). There were also proteins with the same trend but slightly with less significance, such as receptor tyrosine kinase‐like orphan receptor ROR1‐ and p53‐related protein kinase TP53RK (also known as PRPK), which are clearly associated with tumor development and metastasis.^49^, ^50^ If the binding with small molecules affects their function, it may dramatically regulate cellular behavior. However, ROR1 has only 3% coverage with two peptides, and the peptide segment that better reflects the signal to protein quantification differences was the ROR2 homologous peptide segment. Therefore, more sensitive methods or materials with highly expressed ROR1 cell lines are needed for confirmation.

**FIGURE 3:**
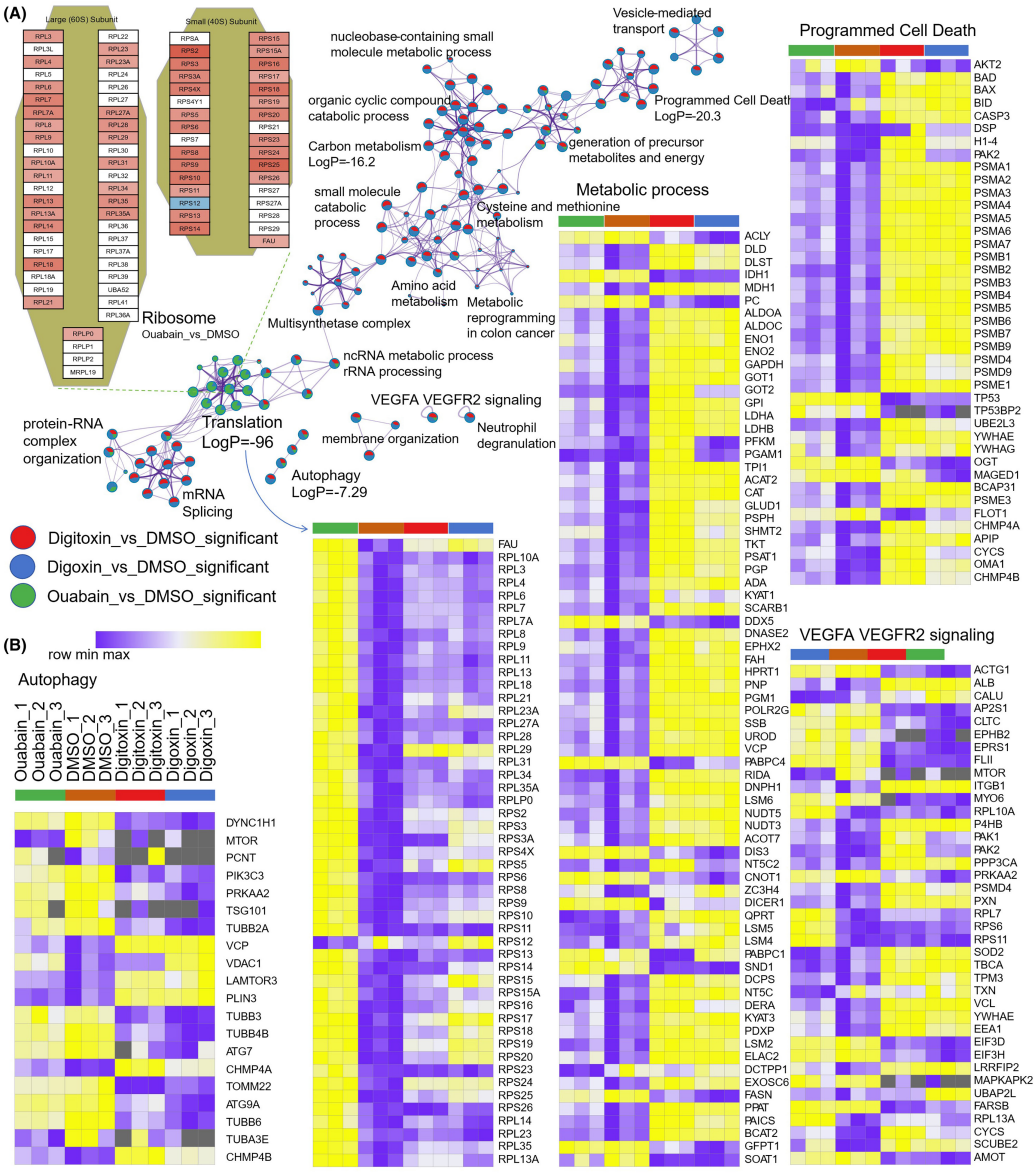
Enrichment analysis for DSPs of CGs lysate‐based PISA targets. (A) Network of enriched terms represented as pie charts, where pies are color‐coded based on the identities of the gene lists. The protein composition of ribosome subunits were highlighted and colored by fold change of comparison between ouabain and DMSO. (B) The heatmap shows the *z*‐scores original data of the enriched terms by row.

Proteins engaging with small molecules typically experience a broad spectrum of functional shifts.^51^, ^52^, ^53^, ^54^, ^55^ Here, Metascape^39^ was used to perform pathway and process enrichment analysis on these DSPs which were regarded as targets, and the results showed that these proteins were enriched in key pathways and process terms, including translation or ribosome, autophagy, programmed cell death, VEGFA VEGFR2 signaling, and metabolism‐related terms (Figure 3 and Table S3). These metabolism‐related terms were mainly enriched in digoxin‐ and digitoxin‐targeted proteins including generation of precursor metabolites and energy which clustered electron transport chain, and translation or ribosome terms were mainly enriched in ouabain‐targeted proteins. Thus, CGs may induce cytotoxicity via direct targeting mitochondrial proteins to affect key metabolic processes such as electron transport chain and tricarboxylic acid (TCA) cycle. Besides, pronounced stabilizing impact of ouabain on ribosomes may elucidate a unique mechanism of action.

### 3.3 Validation of targets of CGs by molecular docking

**FIGURE 4:**
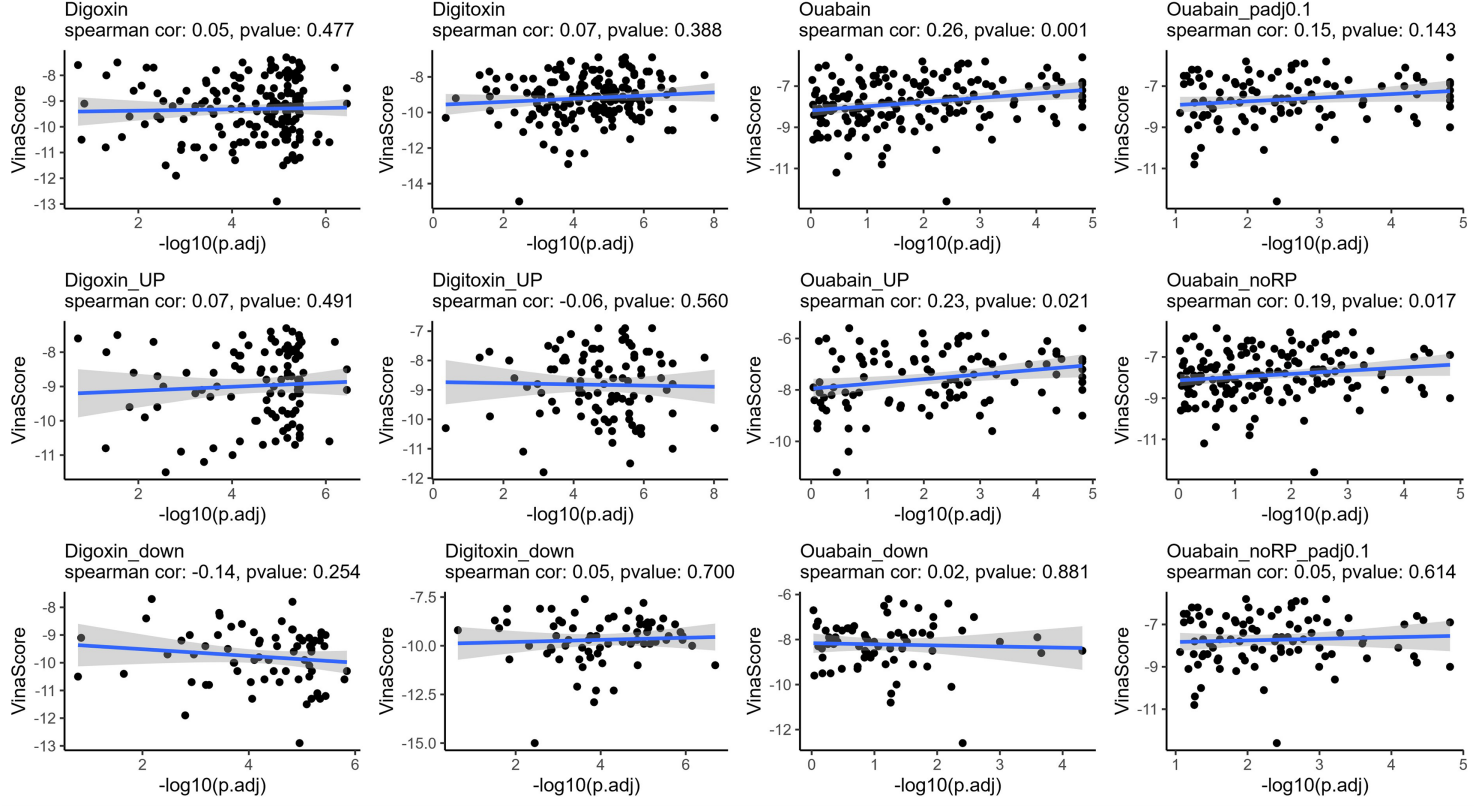
Batch molecular docking to explore the interactions between CGs and targets. The Spearman correlation between affinity score (represented as Vina score) and thermal stability score calculated with‐log10 (pAdj) is shown. And increase or decrease in solubility changed DSPs were drawn separately indicated by “up” for increases and “down” for decreases. “padj0.1” meaning filtering ouabain DSPs by pAdj <0.1 and “noRP” meaning removing ribosomal proteins.

We then utilized CB‐Dock2 for extensive molecular docking to explore the interactions between CGs and DEPs (Figure 4 and Table S4). The majority of DSPs demonstrated a top‐ranked Vina score less than −6, indicating a robustly credible interaction. However, the predicted binding of most ribosomal proteins with ouabain was weaker, suggesting that the binding outcomes may be due to ouabain disrupting the ribosomal complex. Additionally, we investigated the correlation between changes in thermal stability and binding affinity scores. In case of digoxin and digitoxin, no correlation was observed between the extent of thermal stability changes and binding affinity, regardless of whether the solubility of DSPs increased or decreased. The correlation with ouabain was found to be caused by non‐ouabain DSPs. Similarly, after filtering ouabain DSPs and removing ribosomal proteins from the differential proteins, the correlation disappeared.

**FIGURE 5:**
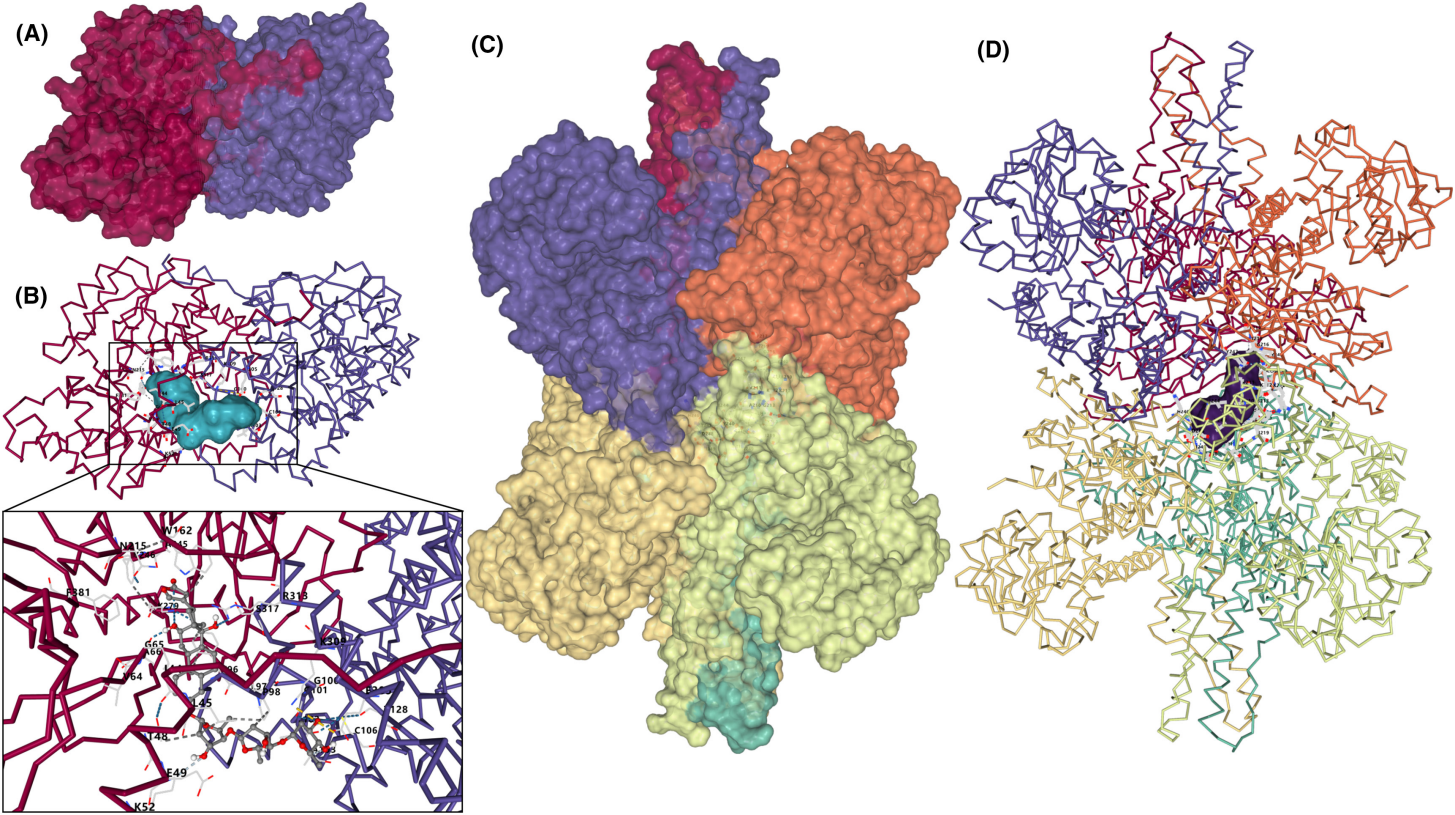
Molecular docking to validate the interactions between digoxin and targets. Docking results of digoxin with GOT2 dimer (A, B) and GLUD1 hexamer (C, D) Backbone diagram of the structure of the GOT2 dimer (B) and GLUD1 hexamer (D), surface diagram of digoxin for batter understand binding conformations and the GOT2 dimer bound digoxin is represented by a ball and stick model. All subunits are colored separately.

We further analyzed the binding affinities and modes of interaction between digoxin and the mature form of GOT2 (Figure 5A, PDB ID, 8skr; resolution, 2.99 Å) and GLUD1 (Figure 5C, PDB ID, 8sk8; resolution, 2.31 Å) (Table 1).^43^ The results showed that digoxin bound to its protein targets through visible hydrogen bonds and strong electrostatic interactions (Figure 5B,D). Moreover, the hydrophobic pockets of each target were occupied successfully by digoxin, and it had high binding energy of −11.2 and − 11 kcal/mol, indicating highly stable binding, and binding of these sites may directly impact its functionality.

**TABLE 1:** Molecular docking to validate the interactions between digoxin and targets. Docking results of digoxin with GOT2 dimer and GLUD1 hexamer.

Targets	CurPocket ID	Vina score	Cavity volume (Å^3^)
GOT2	C2	−11.2	1283
	C5	−10.9	1072
	C1	−10.4	1394
	C3	−8.8	1252
	C4	−8.5	1239
GLUD1	C1	−11	53,566
	C2	−10.6	12,754
	C5	−9.9	1036
	C3	−7.8	2866
	C4	−5.9	2727

### 3.4 Intact‐cell PISA to identify CGs targets and mechanism of action

PISA can also be utilized in the context of living cells to investigate target binding within the cellular environment, allowing for the exploration of natural states of proteins and interactions. With the same data processing protocol as lysate‐based PISA (Figure S3 and Table S5), 744, 815, and 727 DSPs were identified in the digitoxin, digoxin, and ouabain groups, respectively (Table S6). Among these DSPs, the well‐established target of CGs, ATP1A1 was identified among the most significantly destabilized proteins in each group. In addition, all other identified Na+/K + ‐ATPase subunits (ATP1B3 and ATP1B1) were DSPs, albeit with slightly lower confidence levels.

Ouabain has been confirmed to inhibit the SLC16A1‐mediated uptake of lactic acid.^56^ Given the comparable significance of SLC16A1 to ATP1A1, it was strongly suggested that SLC16A1 could be a validated target within the spectrum of CGs. Moreover, the direct binding of digoxin to pyruvate kinase M2 (PKM2) has also been established.^11^ We identified PKM2 as the canonical isoform of PKM in 293T cells and reliably excluded other alternative splicing isoforms (Figure S4). Notably, PKM2 was particularly prominent among the differentially stabilized proteins, especially in the context of digoxin treatment. These findings further validated the reliability of our methodology.

Metascape enrichment analysis showed that DSPs were enriched in some key pathways and process terms, including translation or ribosome, programmed cell death, VEGFA VEGFR2 signaling, and metabolism‐related terms enriched with high confidence (Table S7). Further, intact‐cell PISA underscored the substantial impact of CG compounds on the stability of ribosome, which is completely contrasting to the effects observed in lysate‐based PISA. The enrichment profiles of the three CGs in intact‐cell PISA were strikingly consistent, which may be attributed toward the influence of Na+/K + ‐ATPase or the significant action of other uniform targets. It has been reported that cells treated with CGs exhibited an accumulation in the G2/M phase and depleted intracellular ATP levels.^57^

**FIGURE 6:**
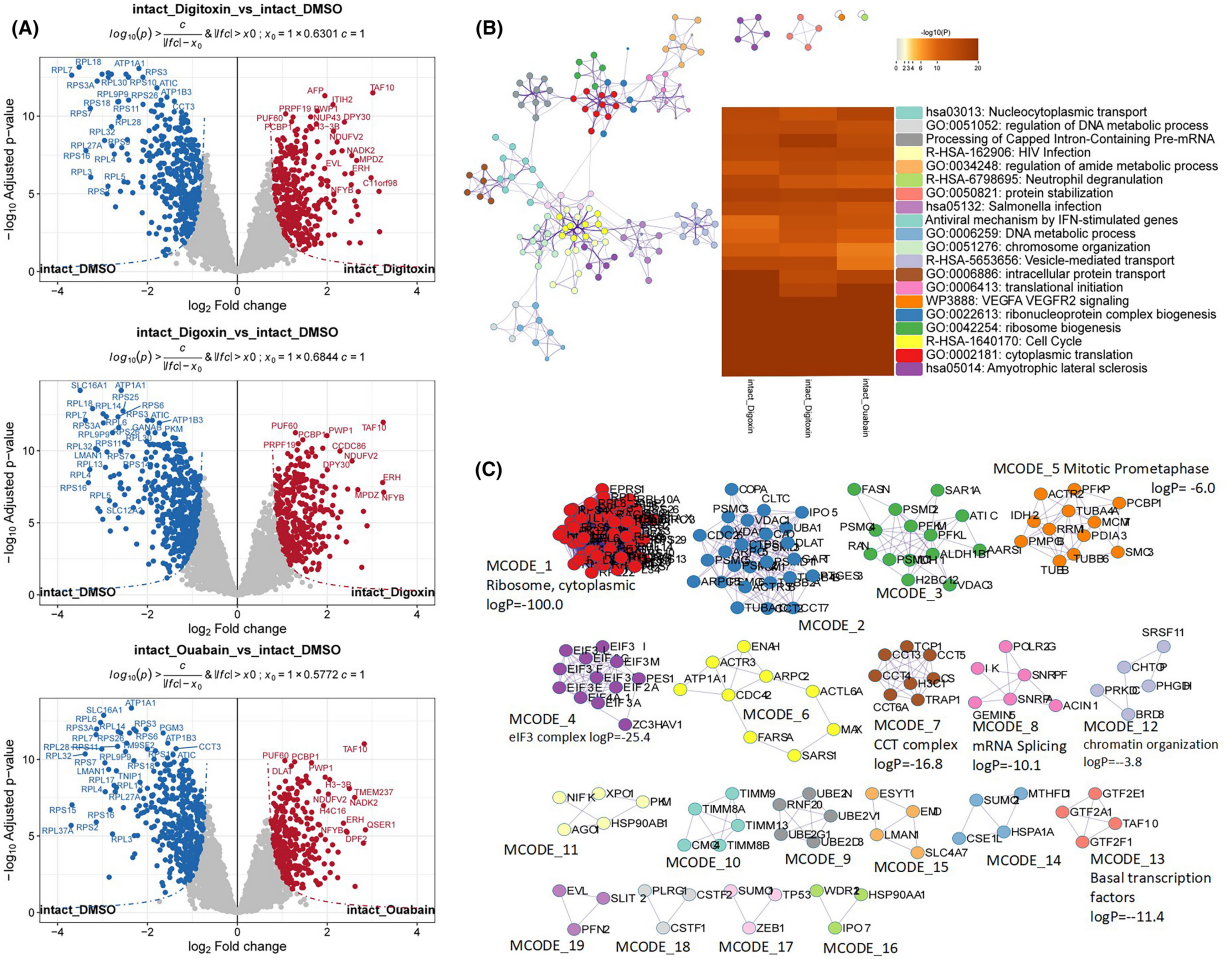
Intact‐cell PISA to identify CGs targets and mechanisms of action. (A) Volcano plots of CGs versus DMSO, DSPs were highlighted red for significant increase in solubility and blue for decrease. (B) Network and heatmap of enriched terms, nodes of network were colored by cluster ID, where nodes that share the same cluster ID are typically close to each other; heatmap colored by *p*‐value. (C) Protein–protein interaction network and MCODE components identified in the gene lists.

Due to the heightened sensitivity of protein complexes to thermal stability, we have focused on the PPI network. The molecular complex detection (MCODE) algorithm has been employed to identify components of the network that are densely connected.^58^ The MCODE networks derived from individual gene lists have been compiled and are presented in the Figure 6C.

The previously described ribosome complex is enriched in MCODE1, while MCODE2 and MCODE3 both show enrichment for the CORUM complex PA700, which, together with the 20S core particle, constitutes the 26S proteasome. Here, the proteins associated with 26S proteasome were examined (Table S7). Proteins related to PA700 complex exhibited a significant difference as compared to others, with the exception of PSMD9, which showed a similar upward trend in the CGs group in the lysate‐based PISA but did not reach up to the significant level. MCODE4 is enriched with the CORUM complex eIF3, and translation initiation factors were analyzed in the present study. The most translation initiation factors showed a downward trend in the CGs treated group, with the notable exception of EIF6, which was significantly up‐regulated. MCODE7 was enriched with the CORUM complex CCT, a large molecular chaperone complex, which showed a significant decrease in stability. Its abundance was very high in lysate‐based PISA, and the CGs group significantly enhanced its identification strength, a pattern consistent with that of the ribosome complex (Table S7).

The intensity heatmaps of these interacting proteins are shown in Figure S5A and Table S7, and both the CCT complex and the PA700 complex were observed to exhibit lower concentrations in intact‐cell PISA compared to lysate‐based PISA. Of note, the PFKM, PFKL, and PFKP kinases showed variations consistent with ATP1A1, as well as nucleoplasm‐located proteins such as RNF113A, HEXIM1, and RAN, a member of the RAS oncogene family (Figure S5A). Given the widespread impact of these interactions, we assessed the proteins for liquid–liquid phase separation (PS) (Figure S5B and Table S8).^59^ Among the 151 proteins categorized under “PS‐self” or “PS‐other” entries, 77 were identified as DSPs in this combined PISA study, highlighting the extensive influence.

Cellular PISA data from docking DSPs were also compiled (Figure S5C and Table S8). While some of these DSPs exhibited similar trends, a considerable number of DSPs displayed opposing trends. This observation underscored the complexity of CETSA and suggested that deconvolution through lysate‐based would be optimal for further elucidation. Furthermore, we observed that more than half of the proteins related to PPIs within intact‐cell are present at concentrations lower than those observed in lysates‐based PISA, and patterns formed by PS or DSPs in lysate‐based did not exhibit a clear trend. This observation reflected the intrinsic state of protein interactions within the cellular environment and further underscored the significance of the notable differences in PPI‐related proteins following treatment with CGs.^60^

### 3.5 Verification of public data

We then compiled and analyzed all chemical–target interactions from Pubchem (https://pubchem.ncbi.nlm.nih.gov/) as well as predictions from Swiss Target Prediction (Figure S5D and Table S8). Those proteins have shown significant changes in intact‐cell or lysate‐based PISA, yet chemical–target interactions from Pubchem include differences in protein expression levels. We manually curated a list of highly likely CGs interacting proteins (Figure S5D and Table S8), indicating that our data have better potential for target discovery.

**FIGURE 7:**
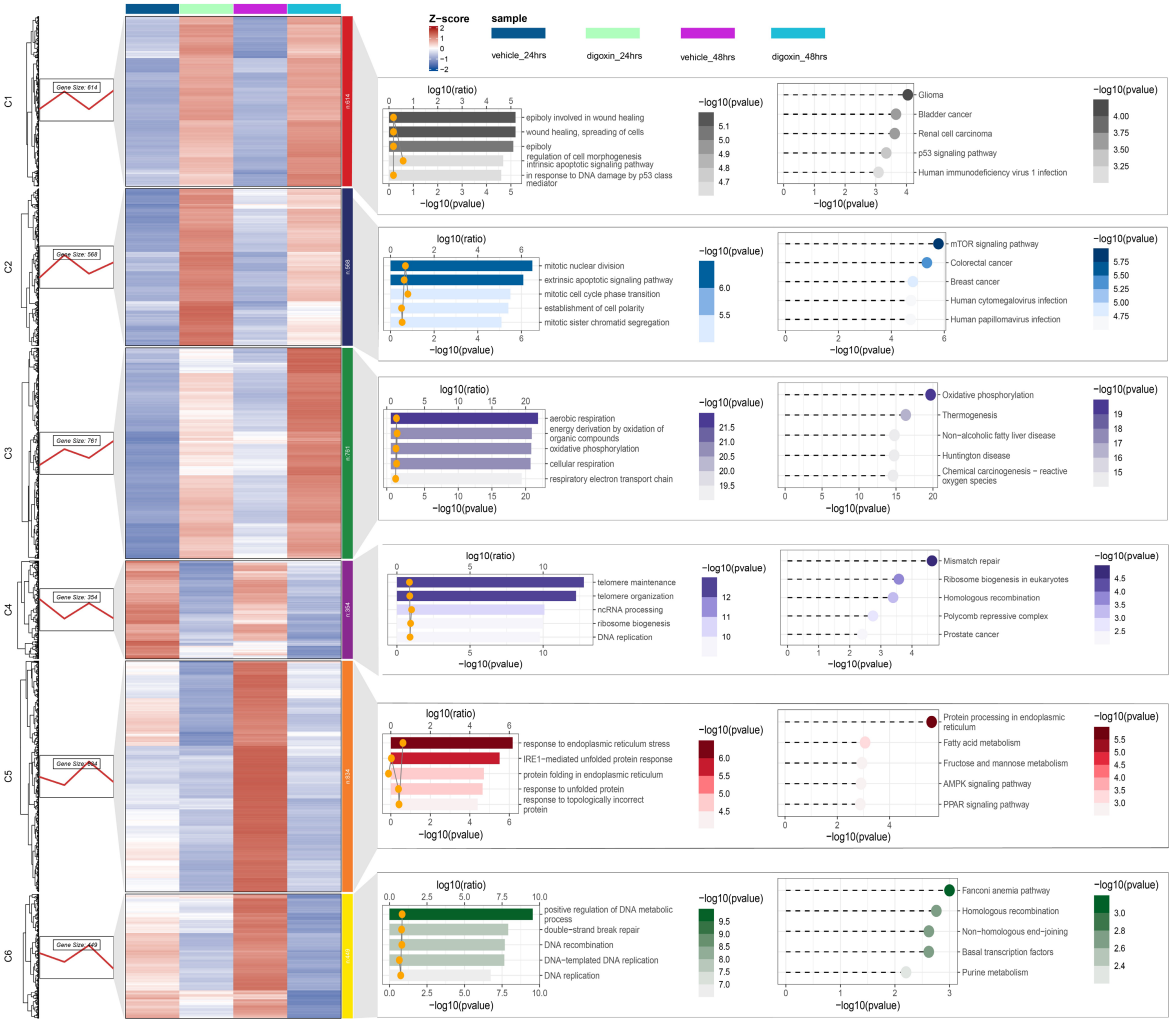
Cluster analysis of reanalyze DEGs in prostate cancer cell lines treated with digoxin. GOBP enrichment analysis results are presented in bar plots and KEGG enrichment presented in bubble plots.

We also conducted a data mining analysis on time‐series transcriptome data and reevaluated the DEGs in prostate cancer cell lines treated with digoxin (Figure S6, Figure 7 and Table S9).^30^ The enrichment analysis of these DEGs revealed that the most significant pathways were aerobic respiration of GOBP and oxidative phosphorylation of KEGG, which were enriched in cluster C3. Other clusters included C1: p53 signaling pathway and glioma; C2: mitotic nuclear division of GOBP, mTOR signaling pathway, and colorectal cancer. Genes associated with these pathways were found to be upregulated by digoxin. Additionally, cluster C4 was enriched for telomere maintenance of GOBP, C5 for protein processing in the endoplasmic reticulum, and C6 for positive regulation of DNA metabolic process, which may be an indicator of tumor suppressor mechanisms involving the p53 and mTOR signaling pathways. Other identified pathways also corresponded to the protein pathways targeted by digoxin. The enrichment analysis results from the trend analysis were all statistically significant with *p*‐values less than 0.001.

In addition, we compiled data from the TOX21 program—a large‐scale screening initiative funded by the National Institutes of Health (NIH)^61^—to assess the outcomes of the three drugs categorized as “active agonists” and “active antagonists” (Table S10). In the TOX21 assays, CGs under investigation were found to impact multiple nuclear receptor pathways, including the retinoid X receptor (RXR), RAR‐related orphan receptor (ROR), estrogen receptor (ER), estrogen‐related receptor (ERR), thyroid‐stimulating hormone receptor (TSHR), androgen receptor (AR), glucocorticoid receptor (GR), and constitutively active receptor (CAR). Notably, E2F‐like factor 1 (E2F1), which is involved in processes such as cell cycle regulation and DNA damage response, was consistently identified as an active antagonist, a finding that aligns with transcriptomic reanalysis results. The mitochondrial toxicity (mitotox) assay results were uniformly indicative of active antagonist activity, suggesting that the compounds target mitochondria.

## 4 DISCUSSION

Recent studies have expanded the medical applications of cardiac glycosides to include the treatment of viral infections, inflammation, cancer, hypertension, and neurodegenerative diseases, with a significant portion of the cancer treatment effects attributed to the modulation of signal transduction pathways via the inhibition of Na+/K + ‐ATPase.^9^, ^10^ The inhibition of the Na+/K + ‐ATPase by cardiac glycosides prevents the effective pumping out of sodium ions from the cell, leading to an increase in intracellular sodium concentration. This elevation in intracellular sodium activates the sodium/calcium exchanger (Na+/Ca^2+^ exchanger), resulting in an increased concentration of intracellular calcium ions (Ca^2+^). The oscillations in intracellular calcium levels triggered by cardiac glycosides, through interaction with the inositol 1,4,5‐trisphosphate receptor (IP3R) on the endoplasmic reticulum, cause transient or repetitive increases in intracellular calcium levels. As a secondary messenger, calcium is involved in various cellular signaling pathways and activates calcium‐dependent transcription factors, such as NF‐κB, which in turn influence cell growth, differentiation, and apoptosis.^10^ The activity of cytoskeleton proteins is also changed, which can affect cell morphology and motility and the integrity of intercellular junction proteins. Na+/K + ‐ATPase can also relay signals through activation of other protein–protein interactions. After binding of cardiac glycosides, the release of the cytoplasmic tyrosine kinase SRC from the complex signalosome begins.^62^ SRC kinase is activated by phosphorylation at Tyr418 and, in turn, activates the proximal epidermal growth factor receptor (EGFR). Activated EGFR then recruits the adaptor proteins SHC, growth factor receptor‐bound protein 2 (GRB2) and SOS until eventually the signal activates the Ras‐RAF‐MAPK (mitogen‐activated protein kinase) cascade.^10^

However, these mechanisms are largely based on theories derived from changes in protein expression levels. It is also crucial to consider the impact of drug‐induced alterations in protein structure and function,^51^, ^52^ a factor that has been infrequently discussed in the context of CGs. Until recently, the discovery of new targets for CGs has been a frequent occurrence.^11^, ^12^ Interestingly, these articles utilized DART and immobilized digoxin affinity enrichment methods, both of these allowed the visual identification of distinct bands on stained gels. This observation provides some evidence that CGs are drugs with a broad range of targets. Digoxin binds to PKM2,^11^ a finding that was also corroborated by our intracellular PISA experiments. The binding of digoxin to PKM2, independent of its kinase activity, leads to chromatin remodeling and a downregulation of hypoxia‐inducible factor‐1α (HIF‐1α) transactivation. This interaction has potential implications for cancer therapy, as PKM2 is highly expressed in most cancer cells, and its overexpression in various tumors has been significantly correlated with patient prognosis.^63^, ^64^ The other target LRP4 not identified in our proteomics, it may not be expressed in 293 T cell lines.

Treatment with digitoxin and ouabain can dissociate circulating tumor cell (CTC) aggregates and has been confirmed to affect the function and localization of tight junction proteins like claudin by increasing intracellular calcium ion concentration, leading to DNA methylation remodeling at key stemness and proliferation‐related loci.^31^ However, the exact mechanisms by which an increase in intracellular Ca^2+^ impacts claudin's function and localization, and how the knockdown of claudin leads to methylation at target sites, are not yet fully understood. Moreover, a concentration of 10 μM digoxin did not demonstrate an effect on dissociating CTC aggregates in initial screenings, which is difficult to explain by a simple increase in Ca^2+^ alone. In this study, intact‐cell PISA identified calcium‐regulated membrane‐binding protein ANXA2, and calmodulin‐like protein 5 (CALML5) as calcium‐associated proteins that may interact directly with CGs, potentially accounting for the varied effects observed. The annexin protein family, which plays a role in various biological processes, is a group of calcium‐dependent phospholipid‐binding proteins closely associated with the occurrence, progression, invasion, and metastasis of cancer.^65^ The Ca^2+^ signaling affected by Na+/K + ‐ATPase activity significantly impacts the annexin protein family.^66^ In our intact‐cell PISA results, ANXA1, ANXA4, ANXA5, ANXA6, and ANXA7 all showed significant decrease in stability in the CGs group. However, ANXA2, the only annexin protein family member that identified as DSP in the lysate‐based PISA, did not reach up to significance in intact‐cell PISA, strongly suggesting that ANXA2 may be bounded by CGs, impairing its normal function. Abnormal expression of ANXA2 is a common feature across many types of tumors, and its expression levels in tumors are closely related to the growth, invasion, and metastasis of cancers such as pancreatic, colorectal, breast, and glioblastoma.^67^ The ANXA2 gene can interact with various genes and participate in multiple signaling pathways, regulating the proliferation cycle of tumor cells.^65^, ^66^, ^67^ Additionally, overexpression of ANXA2 in extracellular vesicles can mediate epithelial–mesenchymal transition (EMT), enhancing the invasiveness of tumor cells.^65^ Furthermore, the expression level of ANXA2 in tumor cells affects the cytoskeletal composition and the plasminogen/plasmin system, playing a crucial role in tumor migration.^65^ CTCs also require the overexpression of ribosomes.^68^ From our lysate‐based PISA results, ouabain may directly affect ribosomal integrity, and there may be other widespread secondary factors other than CGs which have important impact on the translation process.

Tumors sustain abnormal growth and survival by altering cellular metabolism, exhibiting a metabolic shift in cancer cells that favors glycolysis over oxidative phosphorylation, even in the presence of oxygen, a phenomenon known as the Warburg effect.^69^, ^70^ This unique aspect of cancer metabolism presents a vulnerability that can be therapeutically targeted without disrupting the function of nonmalignant cells.^71^, ^72^ Here, lysate‐based PISA identified significant upregulation of DSPs, among which GOT2, GLUD1, and CYCS are crucial for energy metabolism: GLUD1, active in both the mitochondria and cytoplasm, participates in the metabolism of glutamate and is a key enzyme in the TCA cycle.^73^ GOT2, a mitochondrial enzyme that has recently garnered considerable interest in cancer metabolism, is a mitochondrial aminotransferase that reversibly catalyzes the conversion of glutamate and oxaloacetate to aspartate and α‐ketoglutarate.^74^, ^75^ CYCS is a component of the mitochondrial electron transport chain (ETC), functioning within Complex IV. Targeted inhibition of these proteins could potentially affect ATP production, disrupt oxidative phosphorylation, and induce reactive oxygen species (ROS). Furthermore, the δ subunit of the mitochondrial ATP synthase, ATP5F1D, identified as a DSP in cellular experiments, may reflect mitochondrial dysfunction. Indeed, systematic changes in the concentrations of energy‐related metabolites can account for the extensive consistent changes observed in cellular experiments, particularly as the depletion of ATP can cause ribosomal stalling, making intact ribosomal complexes more prone to aggregation.

We have also taken note of the solute carrier (SLC) protein family, particularly SLC16A1, SLC7A1, and SLC7A5 in cellular experiments, as well as SLC25A1, SLC44A1, and SLC44A2 in extracellular experiments. Notably, SLC25A1 was identified as a DSP in both types of experiments, with more pronounced fluctuations in vivo. It encodes the mitochondrial citrate/isocitrate carrier, a transport protein located on the inner mitochondrial membrane responsible for the translocation of citrate and isocitrate. SLC25A1 plays a pivotal role in the TCA cycle, as it facilitates the transport of intermediate metabolites into and out of the mitochondria. Reports have indicated that downregulation of SLC25A1 expression can significantly inhibit the proliferation of colorectal cancer cells by suppressing the progression of the G1/S cell cycle phase and inducing apoptosis, as observed in both intact‐cell and lysate‐based PISA.^76^ SLC25A1 has also been implicated in the reprogramming of energy metabolism: under normal conditions, it promotes CRC growth by increasing de novo fatty acid synthesis. Under metabolic stress, it protects CRC cells from apoptosis induced by energy stress by enhancing oxidative phosphorylation. Similarly, lung cancer initiation or cancer stem cells generate energy and survive in a manner dependent on the activity of SLC25A1, which plays a key role in maintaining the mitochondrial pool of citrate and redox balance in CSCs.^77^, ^78^ Its inhibition leads to the accumulation of reactive oxygen species, thereby suppressing the self‐renewal capacity of cancer stem cells. Furthermore, resistance to treatments such as cisplatin or EGFR inhibitors in tumors derived from different patients is mediated by mitochondrial activity and the induction of a stem‐like phenotype via SLC25A1.^78^ Therefore, the combination of SLC25A1 inhibitors with cisplatin or EGFR inhibitors represents a synthetic lethal approach, and there is also a sensitization phenomenon with the combination of digoxin and cisplatin. Additionally, ouabain has been confirmed to inhibit the SLC16A1‐mediated uptake of lactate,^56^ a key product of glycolysis and a process favored by cancer cells due to the Warburg effect. The consistent high‐confidence thermotolerance alterations observed in all CGs strongly suggest that SLC16A1 is a novel, identified target for CGs. Given the central role of SLC16A1 in cancer cell metabolism,^79^ it may serve as a potential target for cancer therapy.

Recent studies have confirmed that ouabain targets complexes of the ETC to inhibit mitochondrial oxidative phosphorylation, leading to a significant decrease in intracellular ATP levels.^57^ The supplementation of ATP (100 μM) can block the activation of AMPK induced by ouabain.^57^ This reliably demonstrates that AMPK, as a key regulator of cellular energy homeostasis, is activated under conditions of energy stress to initiate downstream pathways and further mediate the induction of autophagy and death through the mTOR signaling pathway in specific cell types. This contrasts with the traditional view that CGs, under the trigger of the RAS‐RAF‐MEK‐ERK and PI3K‐AKT‐mTOR signaling transduction pathways, cause proliferating cells to import nutrients and amino acids.^10^ This stimulates mammalian target of rapamycin complex 1 (mTORC1) and induces transcriptional reprogramming through the activation of MYC and other transcription factors,^10^ which observed through the trend analysis of transcriptome.

In the present study, intact‐cell PISA have also identified the bifunctional purine biosynthesis protein ATIC as DSP. ATIC is a key enzyme in the purine biosynthetic pathway, which is often upregulated in cancers and catalyzes the final two critical steps in purine synthesis.^80^, ^81^ CGs that target ATIC may also hold potential in this pathway for cancer treatment. Indeed, methotrexate, aminopterin, and pemetrexed are commercial chemotherapeutic agents that target the purine biosynthetic pathway, inhibiting the de novo synthesis of purine and pyrimidine nucleotides. Additionally, in lysate‐based PISA, a significant decreased stability in ALDH18A1 was observed, with the highest confidence in response to digoxin and digitoxin. ALDH18A1 is a mitochondrial enzyme belonging to the aldehyde dehydrogenase family. It has various functions within the organism, including catalyzing the conversion of glutamate to glutamic semialdehyde, which is then further converted into proline, ornithine, and arginine. The protein encoded by the ALDH18A1 gene is highly conserved across vertebrates and has evolved from the bacterial proBA operon encoding glutamine kinase (G5K) and glutamyl phosphate reductase (GPR), resulting in the bifunctional ALDH18A1 gene.^82^ ALDH18A1 is associated with the onset and progression of neuroblastoma and may influence tumor cell proliferation and self‐renewal through a positive feedback mechanism with the MYCN gene.^83^

Moreover, ATIC‐associated de novo purine synthesis is critically involved in arterial disease.^84^, ^85^ Mutations in the ALDH18A1 gene have been associated with certain diseases, such as cutis laxa and spastic paraplegia. Symptoms of these conditions may include dermatological manifestations like sagging of the skin, joint laxity, and cataracts, which are indicative of premature aging, as well as intellectual disability and progressive neurodegeneration.^86^ In addition, proteins from the charged multivesicular body protein (CHMP) family, specifically CHMP4A, CHMP4B, and CHMP5, have all shown significant differences, particularly in relation to enveloped viruses. Interferon signaling has also been notably enriched within the cell. These DSPs may play a role across various diseases and could account for well‐known side effects.

The deployment of chemical proteomic methods, such as TPP, in the earlier stages of drug development is highly beneficial.^13^ This is because it allows for the early detection of potential off‐target effects, which aligns with the strategies for tracing off‐target effects of traditional pharmaceutical molecules. For instance, the off‐target effect of panobinostat on phenylalanine hydroxylase (PAH) has been identified,^87^ and the binding of panobinostat to PAH results in increased cellular phenylalanine and decreased tyrosine levels, which are associated with the side effects of the drug.^87^ Our data provide a possible partial explanation for the well‐known pleiotropic effects of digoxin‐like drugs. Large‐scale experiments based on thermal stability have been discussed and have confirmed the reliability of our approach.^19^, ^20^ However, DSPs we have identified may not necessarily impact protein function; we suggest treating DSPs as mutations, which is the reason for our analysis of pathways and interactions. Of course, some high‐confidence proteins are more likely to be affected by their activity. There is research suggesting that interactions with small molecules can affect protein structure and, consequently functions.^51^, ^52^, ^53^, ^54^, ^55^ We offer this PISA results as potential resources for the polypharmacology of CGs, and further downstream experiments are required to confirm that the binding of CGs indeed alters functional mechanisms. Terwilliger et al. have highlighted the limitations of AlphaFold2, particularly in scenarios that require high‐precision side‐chain modeling, such as small molecule drug docking.^88^ However, CB‐Dock2 improved by integrating a template‐based blind docking module, which empowers users to obtain potential binding sites and binding modes by referring to known protein–ligand structure information.^41^ The absence of a direct correlation between affinity and thermal stability underscores the fact that alterations in thermal stability do not necessarily align with changes in binding affinity. This observation resonates with existing perspectives suggesting that the degree of thermal stability change is primarily attributed to the intrinsic properties of the protein itself.^20^ While the significant improvement in hit rate with AlphaFold3 might reveal new correlations.^89^ It is regrettable to be not open‐sourced and limited to dock with designated molecules. A probe‐based chemical proteomics approach has been used to identify and analyze the interactions between small molecular fragments and proteins.^90^ This approach has analyzed 407 structurally diverse small molecular fragments and employed these data to train machine learning models to predict protein–target interactions. Similar modeling methods could be developed based on the thermal stability of proteins^91^, ^92^, ^93^ or lip‐MS.^15^ These MS‐based chemoproteomics methods are bound to accelerate the understanding of small molecule drug docking, contributing to the foundational theory that aids in the discovery of new drugs and the repurposing of existing drugs.

Despite the valuable insights provided by this study, certain limitations must be acknowledged. The models and methodology used may have inherent limitations and potential biases. Specifically, the metabolism of 293T cells may differ significantly from that of other cancer cell lines, which could lead to discrepancies in the experimental results. Additionally, some aspects of the discussion may be overly speculative and may require further experimental validation.

## 5 CONCLUSIONS

This study highlights the potential of repurposing CGs for cancer therapy, suggesting their potentially interactions with key proteins involved in cellular metabolism, ribosomal function, and mitochondrial activity. The comprehensive PISA dataset provided here serves as a valuable resource for understanding the polypharmacological effects of CGs and supports further investigation into their precise mechanisms of action and potential clinical applications.

## AUTHOR CONTRIBUTIONS

**Wenjie Qin:** Conceptualization (equal); data curation (equal); formal analysis (equal); investigation (equal); methodology (equal); project administration (equal); writing – original draft (lead). **Yinhua Deng:** Data curation (equal); formal analysis (equal); investigation (equal). **Huan Ren:** Formal analysis (equal); investigation (equal). **Yanling Liu:** Writing – original draft (equal). **Ling Liu:** Investigation (equal). **Wenhui Liu:** Investigation (equal). **Yuxi Zhao:** Methodology (equal). **Chen Li:** Investigation (equal). **Zhiling Yang:** Conceptualization (equal); investigation (equal); project administration (equal); writing – original draft (equal); writing – review and editing (lead).

## CONFLICT OF INTEREST STATEMENT

The authors declare no conflicts of interest.

## Supporting information


Figure S1:
Figure S2:
Figure S3:
Figure S4:
Figure S5:
Figure S6:

Table S1:

Table S2:

Table S3:

Table S4:

Table S5:

Table S6:

Table S7:

Table S8:

Table S9:

Table S10:

## Data Availability

The mass spectrometry proteomics data have been deposited to the ProteomeXchange Consortium (https://proteomecentral.proteomexchange.org) via the iProX partner repository9 with the dataset identifier PXD052740.
