# Correction to “Exploring the Anticancer Mechanism of Cardiac Glycosides Using Proteome Integral Solubility Alteration Approach”

**DOI:** 10.1002/cam4.70381

**Published:** 2024-11-06

Qin W , Deng Y , Ren H , Liu Y , Liu L , Liu W , Zhao Y , Li C , Yang Z . Exploring the Anticancer Mechanism of Cardiac Glycosides Using Proteome Integral Solubility Alteration Approach. Cancer Medicine 2024;13(18):e70252. 10.1002/cam4.70252.39350574 PMC11442762

In the first of the affiliations, the text “The First Affiliated Hospital of Hunan Normal University (Hunan Provincial People's Hospital)” was incorrect. This should have read: “Hunan Provincial People's Hospital, (The First Affiliated Hospital of Hunan Normal University)”.

We apologize for this error.

