# Supplementary material for: Exploring the anticancer mechanism of cardiac glycosides using proteome integral solubility alteration approach
Source: Cancer Med. 2024 Sep 30;13(18):e70252. doi: 10.1002/cam4.70252 (PMC11442762; doi:10.1002/cam4.70252)
Supplement: Supplementary file 1 — Figure S1: Figure S2: Figure S3: Figure S4: Figure S5: Figure S6: [file CAM4-13-e70252-s005.pdf]

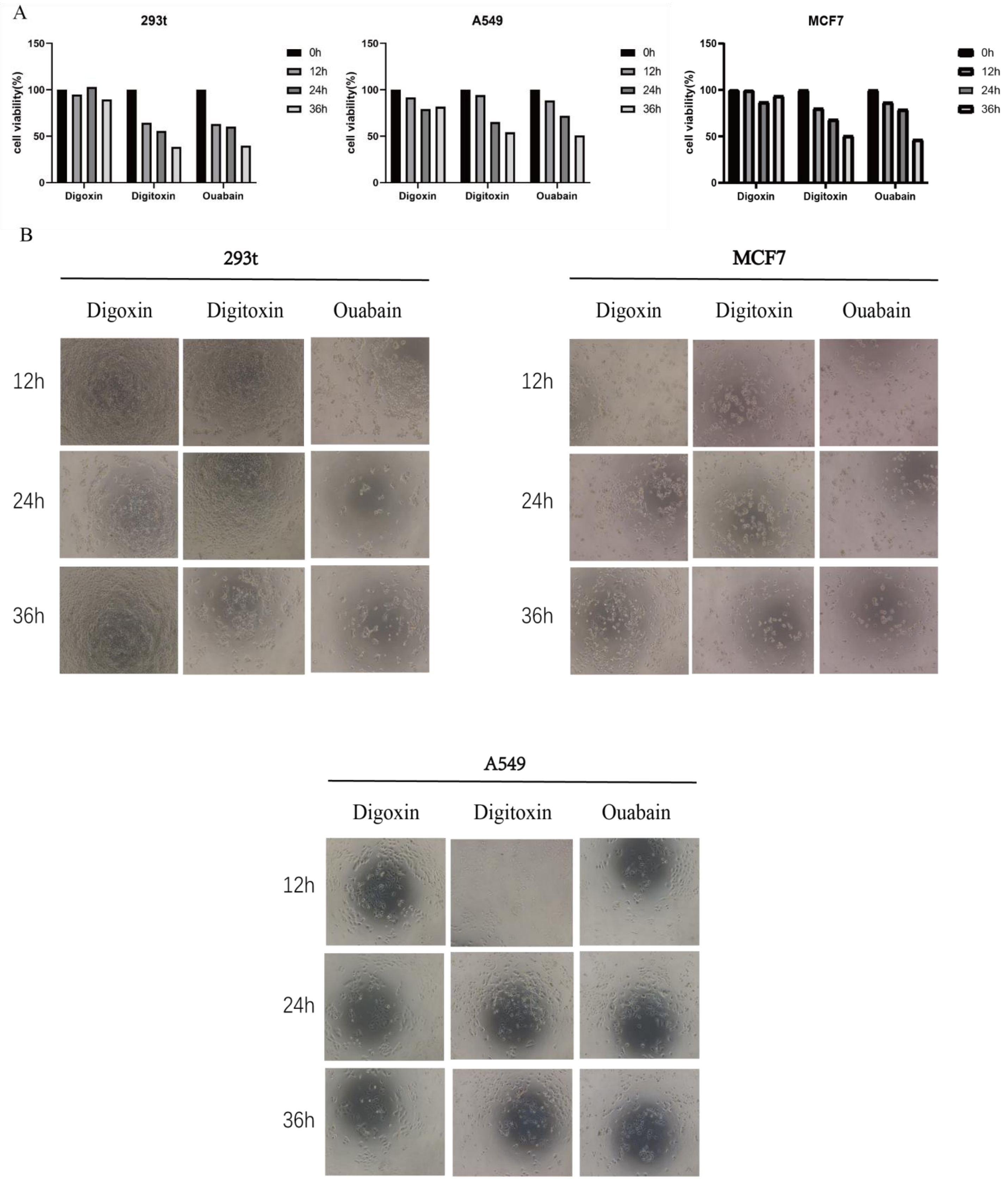

Figure S1. Effects of cardiac glycosides on cell viability in different cell lines. (A). Changes in cell viability percentages of 293t, A549, and MCF7 cell lines at various time points (0h, 12h, 24h, 36h) after treatment. The treatments (40 nM) include digoxin, digitoxin, and ouabain. (B). Morphological changes observed under a microscope in 293t, MCF7, and A549 cell lines at different time points (12h, 24h, 36h) post-treatment with digoxin, digitoxin, and ouabain (40 nM). The images display the trend of cellular morphological changes after drug treatments.

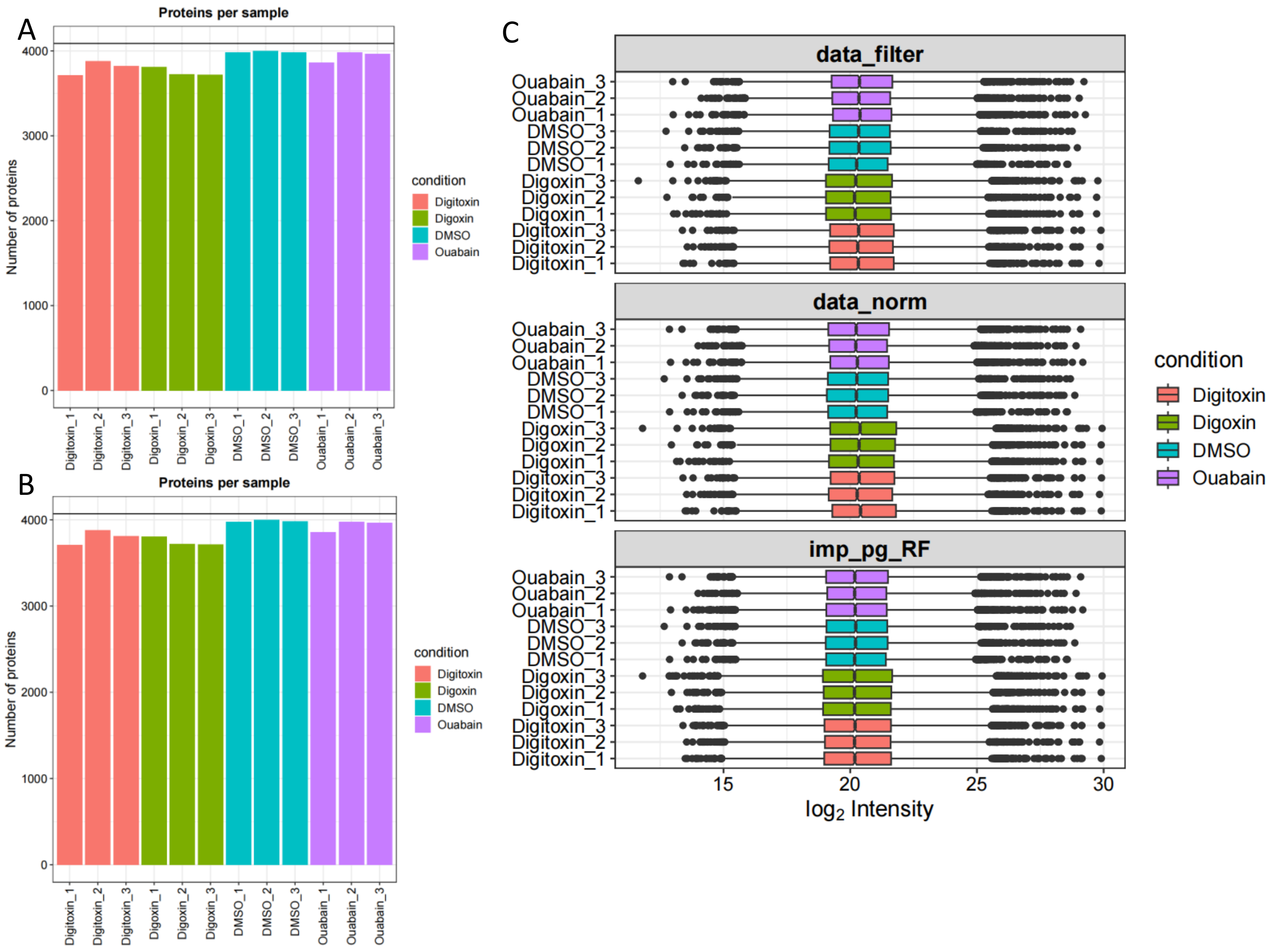

Figure S2. Lysate-based PISA data processing. (A). Barplot of the number of identified proteins per samples. (B). Barplot of the number of identified proteins filtered base on missing number is < = 1 in at least one condition. (C). Visualize normalization by boxplots for all samples before and after normalization and imputation.

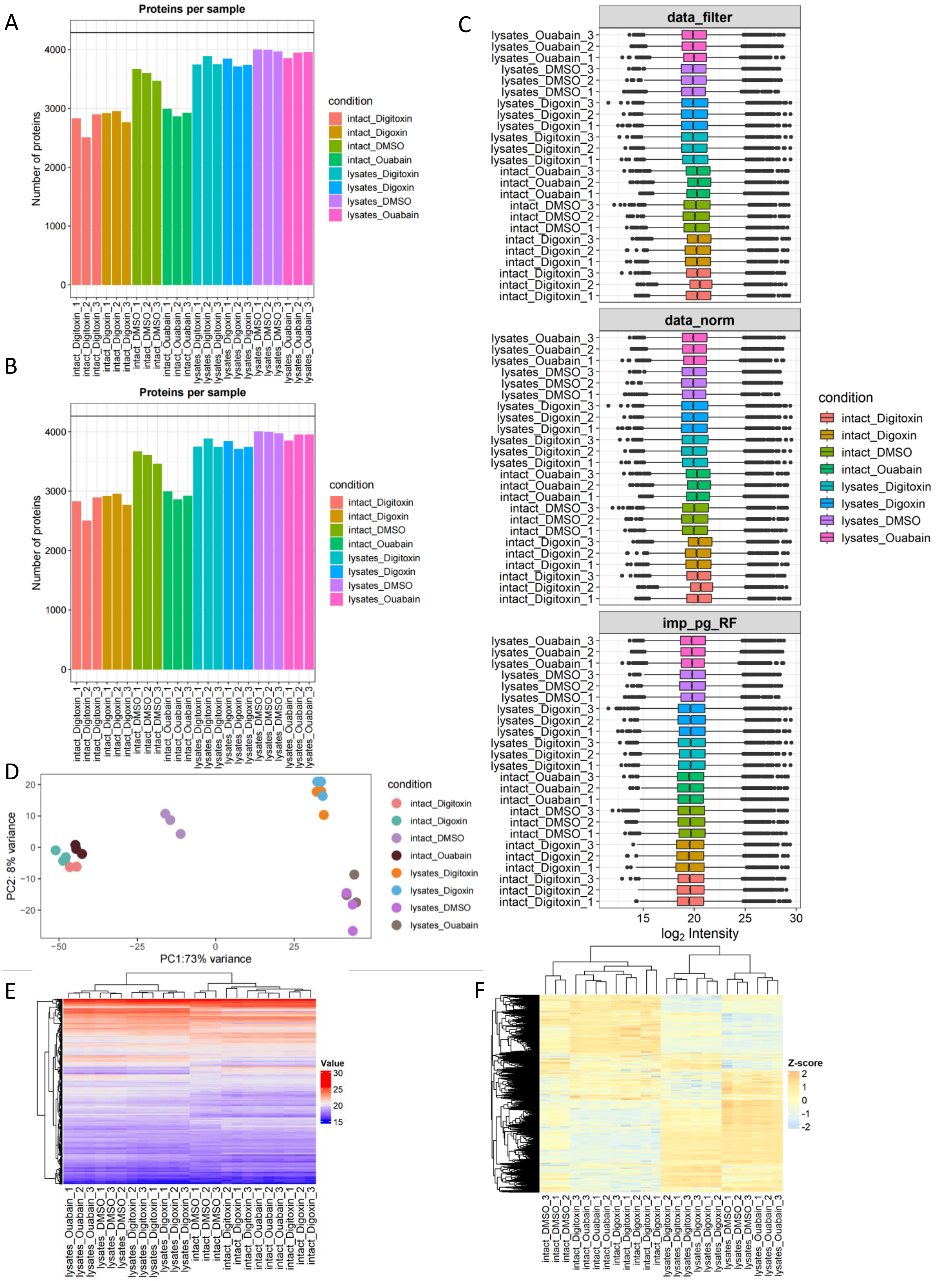

Figure S3. Intact-cell and lysate-based PISA data processing. (A). Barplot of the number of identified proteins per samples. (B). Barplot of the number of identified proteins filtered base on missing number is < = 1 in at least one condition. (C). Visualize normalization by boxplots for all samples before and after normalization and imputation. (D). PCA analysis of all PISA groups. (E). Heatmap combined with dendrogram to show clustering of samples by processed quantitative proteomics intensity values. (F). The heatmap shows the z-scores of the processed quantitative proteomics data by row.

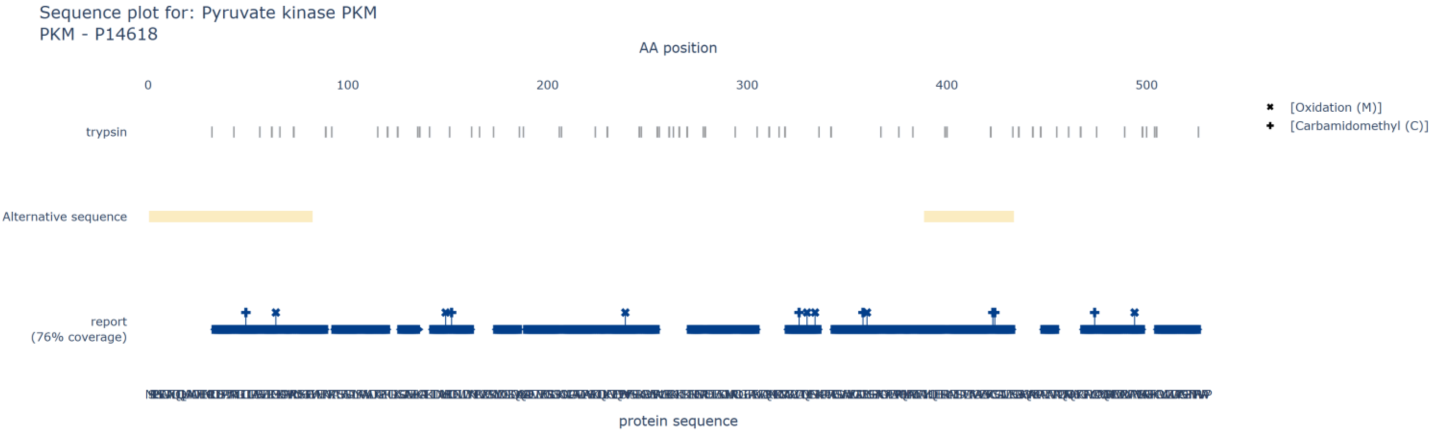

Figure S4. Sequence plot for PKM2. PKM2 as the canonical isoform of PKM in 293T and reliably excluded other alternative splicing isoforms.

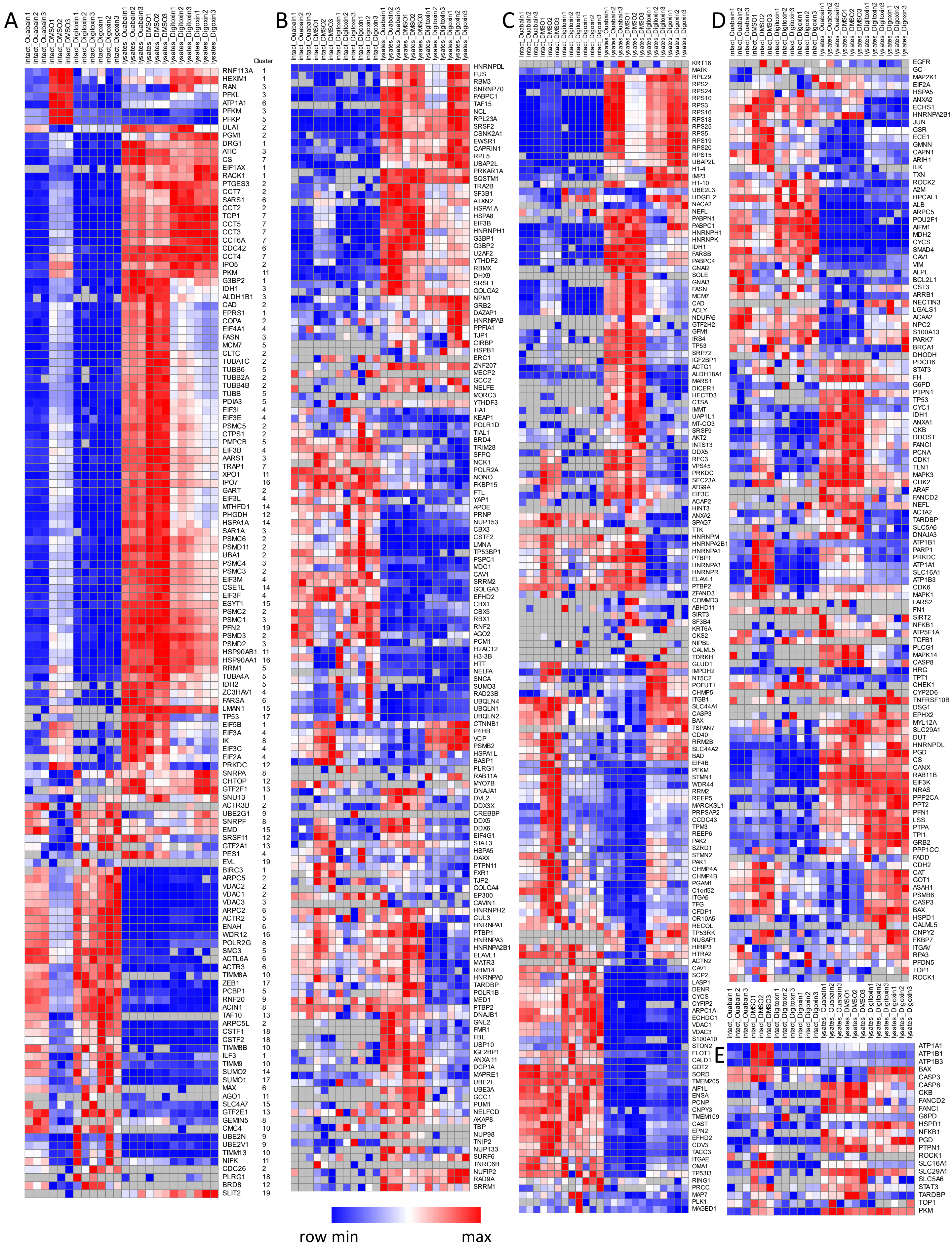

Figure S5. Heatmaps of protein sets. The heatmap shows the z-scores of the processed quantitative proteomics data by row. And rows were clustered by pearson correlation. (A). Heatmap of the intact-cell PISA identifed interacting proteins. The MCODE network clusters have been presented. (B). Heatmaps of proteins categorized under liquid-liquid phase separation. (C). Heatmaps of lysate-based DSPs analyzed by molecular docking. (D). Heatmap of proteins interacting with CGs, compiled from Pubchem or predicted by Swiss Target Prediction. (E). Heatmap of proteins highly likely interacting with CGs, manually curated from Pubchem or predicted by Swiss Target Prediction.

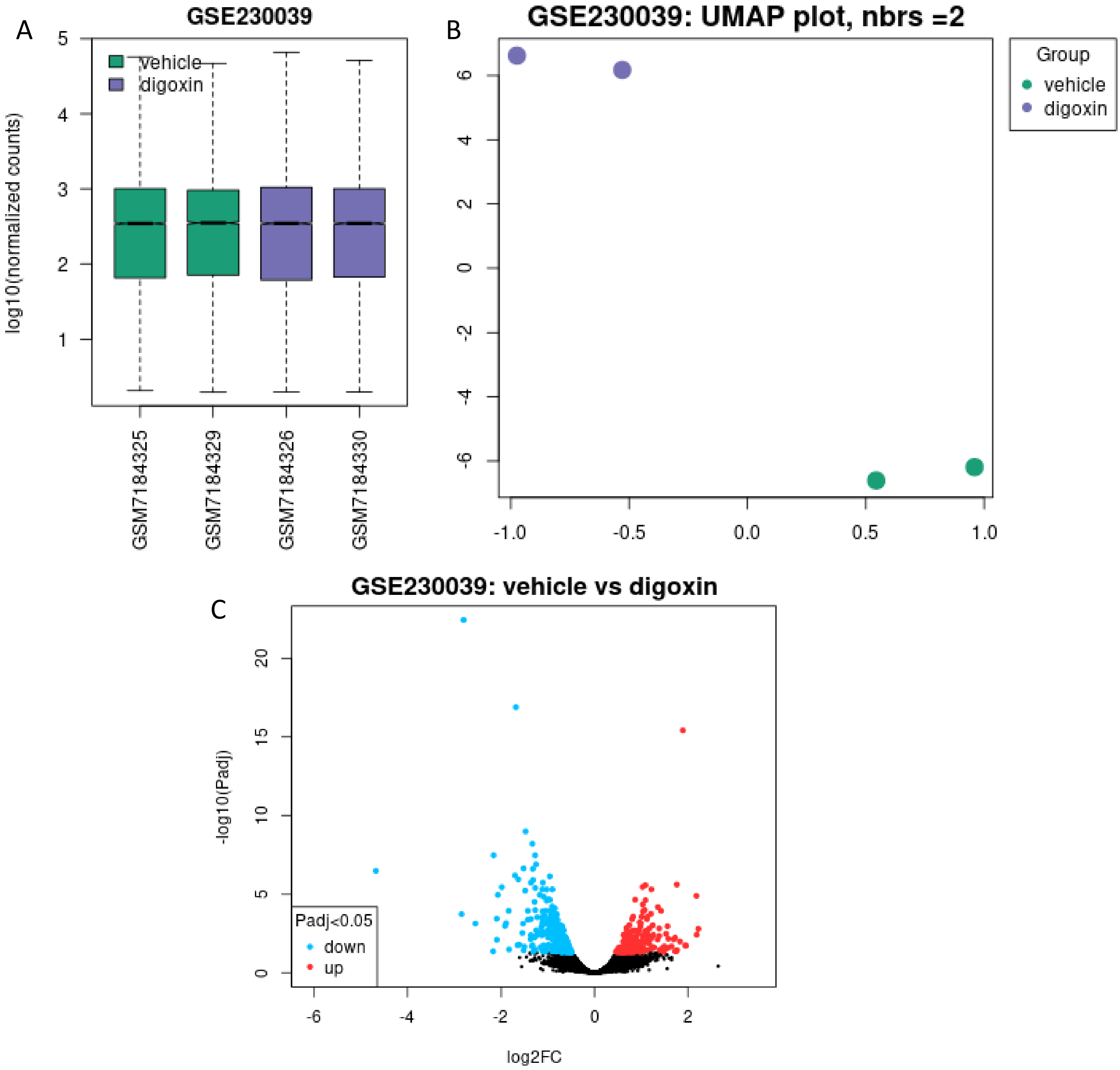

Figure S6. Re-analyse of DEGs using GEO2R in C4-2B cells treated with Digoxin. (A). Boxplot of the distribution of the selected samples. The plot shows RNA-seq data after log transform and normalization. (B). Uniform Manifold Approximation and Projection (UMAP) is used for visualizing how Samples are related to each other. (C). A volcano plot displays statistical significance (-log10 P value) versus magnitude of change (log2 fold change). Highlighted genes are significantly differentially expressed at a default adjusted p-value cutoff of 0.05 (red = upregulated, blue = downregulated).
